# Cardiovascular Mortality Risk among Patients with Gastroenteropancreatic Neuroendocrine Neoplasms: A Registry-Based Analysis

**DOI:** 10.1155/2021/9985814

**Published:** 2021-06-26

**Authors:** Shenghong Sun, Wei Wang, Chiyi He

**Affiliations:** Department of Gastroenterology, Yijishan Hospital of Wannan Medical College, Wuhu, Anhui Province, China

## Abstract

**Background:**

This research is aimed to explore mortality patterns and quantitatively assess the risks of cardiovascular mortality (CVM) in patients with primary gastroenteropancreatic neuroendocrine neoplasms (GEP-NENs).

**Methods:**

We extracted data from the Surveillance, Epidemiology, and End Results (SEER) database for patients diagnosed with GEP-NENs between 2000 and 2015. The standardized mortality ratio (SMR) and the absolute excess risk were obtained based on the reference of the general US population. The cumulative incidence function curves were constructed for all causes of death. Predictors for CVM were identified using a multivariate competing risk model.

**Results:**

Overall, 42027 patients were enrolled from the SEER database, of whom 1598 (3.8%) died from cardiovascular disease (CVD). The SMR for CVM was 1.20 (95% CI: 1.14-1.26) among GEP-NEN patients. The cumulative mortality of CVD was the lowest among all causes of death, including primary cancer, other cancer, and other noncancer diseases. Furthermore, age at diagnosis, race, Hispanic origin, sex, marital status, year of diagnosis, grade, education level, region, SEER stage, primary site, surgery, and chemotherapy were identified as independent predictors of CVM in GEP-NEN patients.

**Conclusions:**

GEP-NEN patients have a significantly increased risk of CVM relative to the general population. Cardioprotective interventions might be considered a preferred method for these patients.

## 1. Introduction

Neuroendocrine neoplasms (NENs) are a collection of fairly rare neoplasms also called “carcinoids” due to their heterogeneous and indolent clinical nature [1]. Gastroenteropancreatic neuroendocrine neoplasms (GEP-NENs) are the most common type, constituting two-thirds of NENs [2]. Over the past 40 years, the incidence of GEP-NENs has grown steadily, with an increase of 3.65 times in the United States and 3.8-4.8 times in the UK [3]. The recently reported annual age-adjusted incidence of GEP-NENs is approximately 3.56/100,000 in the United States and 4.60/100,000 in the United Kingdom [4, 5]. Advancements in diagnostic endoscopy, greater physician awareness, and improvements in cancer treatments have led to considerable improvements in the outcome of GEP-NEN patients, with 3- and 5-year overall survival rates of 79.4% and 74.7%, respectively [6, 7].

A previous study reported that cardiovascular mortality (CVM) increased by 21.1% from 2007 to 2017 globally [8]. In 2016, approximately 17.9 million people died of cardiovascular disease (CVD) globally, accounting for 31% of total global deaths, while approximately 9 million deaths were caused by cancer [9, 10]. In 2017, Kochanek et al. reported that 647457 deaths were due to diseases of the heart while 599108 deaths were due to primary malignant neoplasms in the United States [11].

As life expectancy increases and mortality rates due to cancer decrease, other causes of death have become more prevalent; as such, CVD is one of the main mortality causes of noncancer death [12]. Several studies have assessed CVM in cancer patients: Gaitanidis et al. and Felix et al. demonstrated that patients with colorectal cancer and endometrial cancer have an 11.7- and 8.8-fold higher risk of CVM than the general population, respectively [13, 14]. Fang et al. concluded that the risk of prostate cancer patients developing CVM in the first month and 7-12 months after diagnosis is 2.05- and 0.92-fold that of the general population, respectively [15]. Weberpals and colleagues have shown that the risk of CVM for breast cancer patients is 0.84 times that of the general population [16]. In summary, the risk of CVM varies significantly in cancer patients depends on the primary site and time after diagnosis compared with the general population.

To our knowledge, no reports in the literature have focused on CVM in patients with GEP-NENs. Therefore, we comprehensively described the risk assessment and patterns for causes of death and identified independent predictors for CVM in GEP-NEN patients in this study. Our findings may help to establish more targeted surveillance strategies and preventative measures for CVD in GEP-NEN patients.

## 2. Materials and Methods

### 2.1. Data Source

We extracted data from patients with primary GEP-NENs between 2000 and 2015 in the Surveillance, Epidemiology, and End Results (SEER) database using the SEER∗Stat software (version 8.3.6) [17]. The SEER database, which includes incidence, survival, and mortality data, is a system of population-based cancer registries sponsored by the National Cancer Institute covering approximately 27.8% of the total US population (based on the 2010 census) [18]. Information on mortality from a reference cohort (representing the general US population) reported in the National Vital Statistics System can also be obtained through the SEER program [19]. Ethical approval of this publicly available information provided by the SEER program was not required.

### 2.2. Study Population

**Figure 1:**
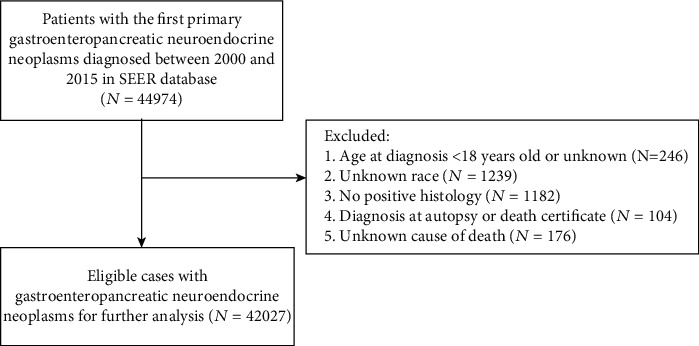
Flowchart of the enrolled patients according to inclusion and exclusion criteria.

Patients histologically diagnosed with GEP-NENs at the first primary tumor aged ≥18 years were retrieved from the SEER database. The following International Classification of Diseases for Oncology, third edition (ICD-O-3) histological codes were used: 8013, 8041-8044, 8150-8153, 8155, 8156, 8240-8046, and 8249. Primary site codes were used for the stomach (C16.0-C16.9), small intestine (C17.0-C17.9, C24.1), appendix (C18.1), colon (C18.0, C18.2-C18.9), rectum (C19.9, C20.9), and pancreas (C25.0-C25.9). Patients with a diagnosis at autopsy or death certificate only and those with incomplete data on certain variables (race, age, and cause of death) were excluded (Figure 1).

The main outcome of interest was CVM, defined by the six causes of death in the SEER database (International Classification of Diseases, 10th Revision [ICD-10] codes): diseases of heart (I00-I09, I11, I13, I20-I51), hypertension without heart disease (I10, I12), cerebrovascular diseases (I60-I69), atherosclerosis (I70), aortic aneurysm and dissection (I71), and other diseases of arteries, arterioles, and capillaries (I72-I78) [20].

### 2.3. Study Variables

Data are presented as the mean ± standard deviation (SD) or median and interquartile range (IQR) for continuous variables and number (percent) for categorical variables. The variables included in this study included age at diagnosis, attained age, year of diagnosis, sex, SEER stage (localized, regional, and distant), race, Hispanic origin, marital status, grade (well differentiated as grade I, moderately differentiated as grade II, poorly differentiated as grade III, and undifferentiated as grade IV), region (Midwest, West, South, Northeast), education level, mean household income, histologic subtype, primary site, surgery, chemotherapy, radiotherapy, and cause of death and survival time. Since there are no personal data on education level or household income in the SEER database, we used the US Census data (2000) to obtain county-specific information on average educational level and household income [15]. Survival time refers to the interval from the diagnosis of cancer to the death of the patients due to any cause or the last date of available survival information [21].

### 2.4. Statistical Analysis

The relative risk of CVM for GEP-NEN patients was compared to all US residents and presented as the standardized mortality ratio (SMR) [22]. The SMR is the ratio of the observed number to the expected number of CVMs [20, 21]. Expected numbers were calculated by multiplying the mortality rate in the reference cohort by the person years (PYs) in the cancer cohort [23]. The absolute excess risk (AER, per 10,000 PYs) was calculated as follows: AER = [(observed deaths − expected deaths)/PYs of observation] × 10,000 [20, 21]. CVM was described as the primary event of interest, while competing events refer to death due to primary cancer, other cancer, and other noncancer causes. The crude cumulative incidence function (CIF) was used to express the probability of developing primary and competing events using the Fine-Gray competing risk model [24, 25]. Multivariate competing risk survival analyses were performed to identify independent predictors of CVM. Data analyses were performed by the R software (version 3.6.3). All tests were 2-sided, and a *P* value < 0.05 signified statistical significance.

## 3. Results

### 3.1. Patient Characteristics

A total of 42027 qualified GEP-NEN patients were included in subsequent analyses. The mean age at diagnosis was 58.57 ± 13.74 years, and the median follow-up time was 54 (22-103) months. The majority of patients were White (74.2%), non-Hispanic (88.2%), married (56.4%), aged ≥50 years (77.6%), had only one neoplasm (88.3%), lived in the Western region (48.8%), and had localized tumor stage (53.3%). The proportion of female patients (21,281 cases, 50.6%) was similar to that of male patients (20,746 cases, 49.4%). The most common primary site was the rectum (30.0%), followed by the small intestine (27.8%) and pancreas (14.1%). Histologic types for GEP-NENs consisted of neuroendocrine tumors (74.9%) and neuroendocrine carcinomas (25.1%). A total of 32265 (76.8%) patients underwent surgery, 4337 (10.3%) patients received chemotherapy, and only 985 (2.3%) patients underwent radiotherapy. Among 42027 patients, 1598 (3.8%) patients died of CVD, with the main cause being diseases of heart (75.3%), followed by cerebrovascular diseases (16.9%) and hypertension without heart disease (4.2%). The baseline characteristics are detailed in Tables 1 and 2.

### 3.2. Standardized Mortality Ratio and Absolute Excess Risk

The SMR for CVM was 1.20 (95% CI: 1.14-1.26), and the AER was 12.63/10,000 PYs in GEP-NEN patients. In the subgroup analyses stratified by different variables, the patients were non-Hispanic; lived in the South, Midwest, and West regions; had an age at diagnosis of ≤39, 40-44, 45-49, 55-59, 65-69, 70-74, and 85+; with an attained age of ≤39, 40-44, 45-49, 50-54, 65-69, 70-74, and 75-79; had a primary site in the stomach, small intestine, or colon; had localized and distant stage; had a latency of 0-1, 2-5, and 6-11 months; and were unmarried, had Grade III/IV disease, with a lower educational level, lower household income, no history of chemotherapy or radiotherapy had significantly elevated SMRs and increased AERs compared with that of the general population, regardless of race, sex, year of diagnosis, subtype, and surgery (Table 1).

The SMRs of deaths from the main causes of CVD in GEP-NEN patients are illustrated in Table 2. Among the six causes, the most significantly elevated SMR was other diseases of arteries, arterioles, capillaries (SMR: 1.74; 95% CI: 1.15-2.54), followed by hypertension without heart disease (SMR: 1.42; 95% CI: 1.10-1.81), cerebrovascular diseases (SMR: 1.21; 95% CI: 1.07-1,36), and diseases of the heart (SMR: 1.18; 95% CI: 1.12-1,25).

### 3.3. Cumulative Mortality of CVD

**Figure 2:**
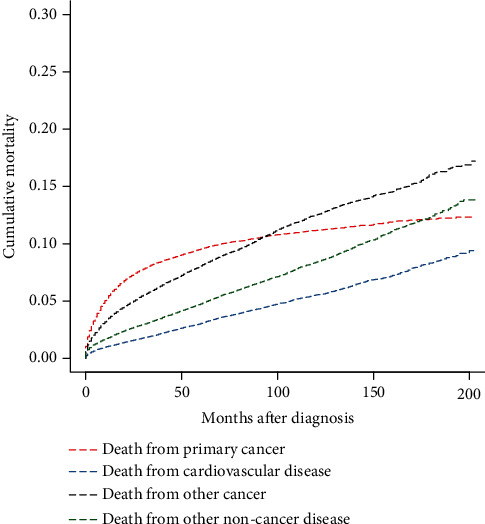
Cumulative mortality for all causes of death in primary GEP-NENs patients.

**Figure 3:**
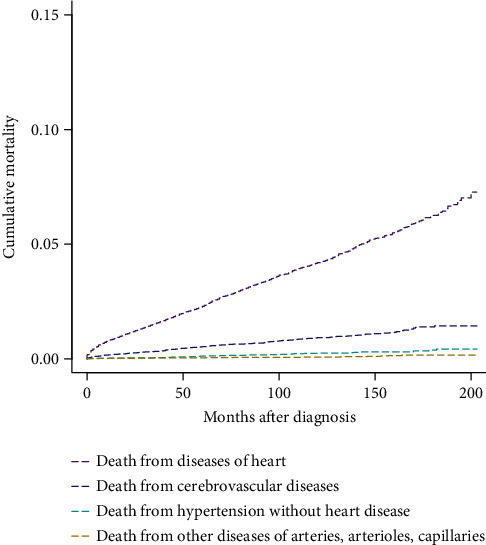
Cumulative mortality for main causes of CVD in primary GEP-NENs patients.

The results of CIF curves for all causes of death in GEP-NEN patients using the Fine-Gray competing risk model are illustrated in Figure 2. The cumulative mortality (CM) of CVD was the lowest among all causes of death. At a follow-up time of 200 months, the CM rates of CVD, primary cancer, other cancer, and other noncancer diseases were 9.4%, 12.3%, 16.9%, and 13.8%, respectively. In the early follow-up period, the highest CM was caused by primary cancer. The CM rates of other cancers and noncancer diseases exceeded that of primary cancer at approximately 90 and 170 months after diagnosis, respectively. As shown in Figure 3, the CM of diseases of the heart was the highest, followed by cerebrovascular diseases and hypertension without heart disease. At a follow-up time of 200 months, the CM rates of diseases of heart, cerebrovascular diseases, hypertension without heart disease, and other diseases of arteries, arterioles, and capillaries were 7.27%, 1.44%, 0.43%, and 0.16%, respectively.

**Figure 4:**
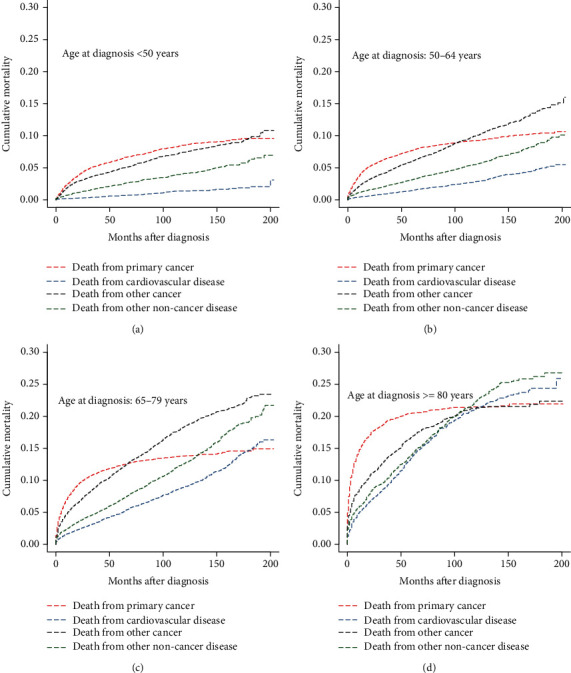
Cumulative mortality for all causes of death in primary GEP-NENs patients stratified by age at diagnosis.

**Figure 5:**
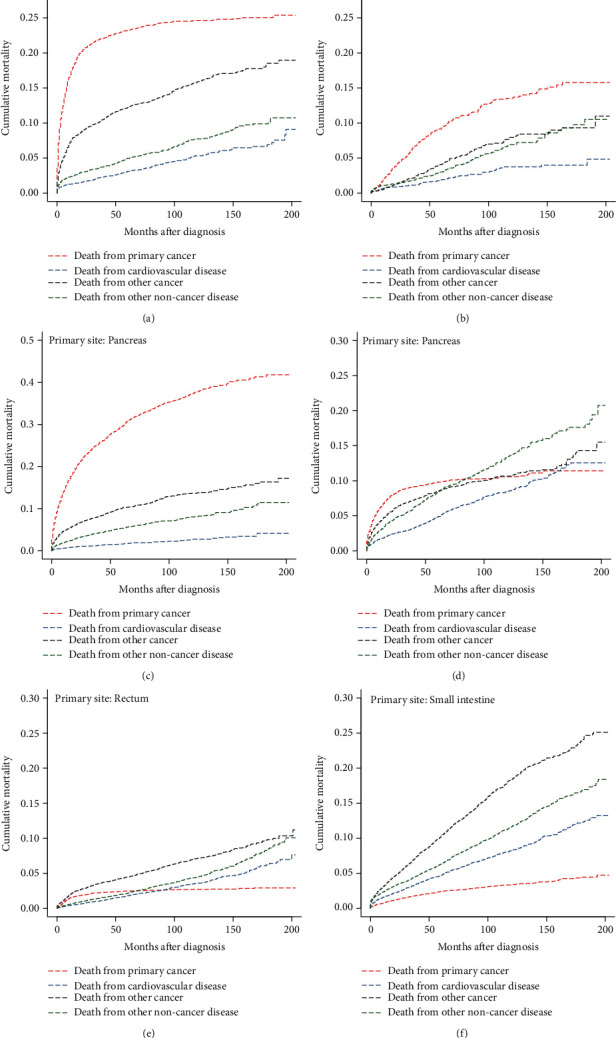
Cumulative mortality for all causes of death in primary GEP-NENs patients stratified by primary site.

In the subgroup analyses stratified by age at diagnosis, we observed that the CM of CVD steadily increased with age at diagnosis (Table 3). The CM of CVD was the lowest of all causes of death in the subgroups of patients aged <50 years (3.1%) and 50-64 years (5.5%) (Table 3, Figures 4(a) and 4(b)). In the subgroups of patients aged 65-79 years and ≥80 years, the CM of CVD exceeded that of primary cancer at approximately 180 months and 120 months after diagnosis, respectively (Figures 4(c) and 4(d)). In the subgroup analyses stratified by primary site, pancreatic and small intestine NEN patients had the lowest (4.12%) and highest (13.26%) CM of CVD, respectively (Table 3). We observed that the CM of CVD was the lowest among all causes of death in the primary tumor site subgroups of the colon (9.09%), appendix (4.84%), and pancreas (4.12%) (Table 3, Figures 5(a)–5(c)). In the primary tumor site subgroups of the stomach and rectum, the CM of CVD exceeded that of primary cancer at approximately 160 months and 90 months after diagnosis, respectively (Figures 5(d) and 5(e)). Interestingly, the CM of CVD in the subgroup of the primary site of the small intestine was higher than that of primary cancer across all follow-up periods (Figure 5(f)).

### 3.4. Predictors of Death from Cardiovascular Disease

We identified indicators associated with CVM in GEP-NEN patients using a multivariate competing risk model (Table 4). We found that the following patient characteristics were independently associated with higher risks of CVM: Black race (HR: 1.307; 95% CI: 1.160-1.472) and non-Hispanic (HR: 1.370; 95% CI: 1.137-1.651), older age (HR: 4.799; 95% CI: 4.313-5.341) and unmarried (HR: 1.562; 95% CI: 1.410-1.173), and no history of surgery (HR: 1.346; 95% CI: 1.188-1.519) or chemotherapy (HR: 1.610; 95% CI: 1.220-2.125). Meanwhile, we found that the following patient characteristics were independently associated with lower risks of CVM: female sex (HR: 0.790; 95% CI: 0.717-0.869), initial diagnosis between 2005 and 2009 (HR: 0.798; 95% CI: 0.717-0.888) and between 2010 and 2015 (HR: 0.575; 95% CI: 0.502-0.659); regional (HR: 0.815; 95% CI: 0.714-0.931) or distant tumor stage (HR: 0.456; 95% CI: 0.382-0.544), grade III/IV (HR: 0.701; 95% CI: 0.533-0.923), college level >25% (HR: 0.798; 95% CI: 0.706-0.902); lived in the Northeast region (HR: 0.813; 95% CI: 0.699-0.945); and primary site in the appendix (HR: 0.698; 95% CI: 0.531-0.918), rectum (HR: 0.550; 95% CI: 0.468-0.646), or pancreas (HR: 0.506; 95% CI: 0.401-0.638).

## 4. Discussion

Multiple studies have confirmed that the risk of CVM among cancer patients varies considerably in different countries. In a population-based study of 21634 adult cancer patients, Ye et al. concluded that the risk of CVM was not significantly different between cancer patients and the general population in Australia (SMR: 0.97; 95% CI: 0.90-1.04) [26]. Oh et al. reported that compared with the general population in Korea, cancer patients have a lower risk of developing CVM (men, SMR: 0.73; 95% CI: 0.70-0.75; women, SMR: 0.83; 95% CI: 0.80-0.87), although they found a 20-fold increase in CVM among cancer patients from 2000 to 2016 [27]. Sturgeon et al. confirmed that the risk of CVM among 28 types of cancer patients was significantly increased compared with that of the general population in the United States, especially in the first year after diagnosis (SMR: 3.93; 95% CI: 3.89-3.97) [8]. A recent study based on the SEER database showed that 1680 (5.6%) NEN patients died from heart diseases and 545 (1.8%) NEN patients died from other CVDs (hypertension without heart disease, cerebrovascular diseases, atherosclerosis, aortic aneurysm and dissection, and other diseases of arteries/arterioles/capillaries), with SMRs of 2.31 (95% CI: 2.20-2.42) and 2.36 (95% CI: 2.17-2.57), respectively [28]. Most NENs are primarily located in the GEP (67.5%) and bronchopulmonary system (25.3%) [29]; however, the 5-year overall survival rates between GEP-NEN (74.7%) and bronchopulmonary NEN (33.7%) patients are significantly different [7, 30]. These findings suggested that NEN patients have various natures and characteristics depending on the primary site. Hence, we focused exclusively on GEP-NENs in the present study.

In this study, we comprehensively assessed the risk of all causes of death among more than 42 thousand GEP-NEN patients from the SEER database and found that the risk of CVM in GEP-NEN patients was 20% higher than that in the general US population (SMR: 1.20; 95% CI: 1.14-1.26). According to the competing risk analyses, we found that the CM of CVD was the lowest among all causes of death, including primary cancer, other cancer, and other noncancer diseases. The CM of diseases of heart ranked first among the main causes of CVD during the follow-up period. In addition, we identified age of diagnosis, race, Hispanic origin, sex, marital status, year of diagnosis, grade, education level, region, SEER stage, primary site, surgery, and chemotherapy as independent predictors of CVM in GEP-NEN patients.

NENs were previously known as carcinoid tumors, in which approximately 50% of patients developed carcinoid syndrome [31]. Approximately 60% NEN patients with carcinoid syndrome develop carcinoid heart disease (CHD), which is characterized by the development of valvular dysfunction, particularly right heart failure [32]. In addition, several studies have found that NEN patients are prone to depression and anxiety [33, 34], which may aggravate the state of cardiovascular physiology [15, 35]. These results may explain the high risk of CVM in patients with NENs to some extent.

In terms of the time after cancer diagnosis, we confirmed that GEP-NEN patients had the highest risk of CVM within the first two months after diagnosis (SMR: 3.64; 95% CI: 3.05-4.30). This finding was similar to previous conclusions reported by Sturgeon et al. and Zaorsky et al. [8, 36]. Moreover, Ye et al. and Fang et al. showed that the recent diagnosis of cancer could be a major psychological stressor and lead to a negative effect on cardiovascular physiology [15, 26, 35]. These results suggested that psychiatric evaluation and psychological support could be indispensable for GEP-NEN patients with a recent diagnosis of cancer. In terms of age at diagnosis, we observed that the CM of CVD steadily increased with the age at diagnosis. This phenomenon resembled previous findings reported by Weberpals et al. and Ye et al. [16, 26]. In general, death from primary cancer was the most common cause of death in cancer patients; however, the CM of CVD exceeded that of primary cancer in patients aged ≥65 during follow-up (Figures 4(c) and 4(d)). These results implied that surveillance efforts should not only include assessment of primary cancer but also control of modifiable risk factors for CVD in elderly cancer patients. In terms of the primary site, we observed that pancreatic NEN patients and small intestine NEN patients had the lowest (4.12%) and highest (13.26%) CM of CVD, respectively. One possible reason was that CHD occurs most frequently in small intestine NEN patients, accounting for 72% [32]. Another plausible explanation was that pancreatic NEN patients had an advanced tumor stage, so they might not have a long enough life expectancy to die of CVD [28, 37, 38], which may explain the lower risk of CVM in patients with grade III/IV (HR: 0.701; 95% CI: 0.533-0.923) or distant tumor stage (HR: 0.456; 95% CI: 0.382-0.544).

Multivariate competing risk analysis was used to identify independent indicators of CVM in GEP-NEN patients in the current study. We found that aged patients at diagnosis were inclined to die due to CVD (HR: 4.799; 95% CI: 4.313-5.341). Interestingly, patients with a younger age at diagnosis (≤39 years) had the highest SMR of 3.20 (95% CI: 1.93-4.99), which was similar to the results reported by Zaorsky et al. [36]. Male patients had a high probability of CVM compared with female patients, as previously reported in colorectal cancer and non-Hodgkin's lymphoma [13, 39]. A plausible reason is that males have worse health behaviors, such as smoking and drinking, which were confirmed as independent risk factors for CVD [40–42]. Our study showed that Black patients were significantly associated with a higher CVM risk than other races. Although patients of different ethnicities had a difference in receiving cancer therapy in the United States, this difference alone cannot explain the discrepancies of cancer patients in terms of death due to noncancer causes [43]. Hence, further investigations on this subject are warranted. Patients who were unmarried showed a propensity to die of CVD in contrast to married patients, as previously reported in non-Hodgkin's lymphoma [39]. A reasonable explanation was that married patients were more likely to feel cared for and encouraged and supported physically and spiritually than unmarried patients [44]. Other studies also revealed that marriage could help to improve cardiovascular, endocrine, immune function, and cancer prognosis [45–47]. Sturgeon et al. reported that individuals with low socioeconomic status were prone to have a high risk of CVM in cancer survivors [8]. In our study, patients with low education levels commonly gave rise to a higher risk of CVM, which was consistent with the results of prior studies [15, 21].

In the present study, a majority (76.8%) of patients underwent surgery, 10.3% of patients received chemotherapy, and only 2.3% of patients received radiotherapy. Notably, multivariate analysis indicated that patients who received chemotherapy had a reduced CVM risk compared with patients who did not receive chemotherapy. This result seemed to be inconsistent with the known cardiotoxic effect of chemotherapy but conformed with the finding reported by Low et al. [28]. A possible reason was that patients who received chemotherapy did not have enough life expectancy to experience CVM events (median survival time: chemotherapy 18 months vs. surgery 61 months). We concluded that patients without surgery had an increased CVM risk compared with patients who received surgery, which was consistent with the results from prior studies [13, 14, 44]. With respect to radiotherapy, a prior study reported that radiation-induced macrovascular damage accelerated age-related atherosclerosis and microvascular damage and reduced capillary density [48]; however, radiotherapy was not an independent predictor for CVM in our study. In the SEER program, radiotherapy was defined as the first-course radiation treatment, but a detailed regimen was lacking. Therefore, further investigation is required to clarify the effect of radiotherapy on the risk of CVM in patients with GEP-NENs.

Limitations still exist in our study. First, some information associated with CVD was not available in the SEER registry, such as comorbidities, smoking and alcohol use, and doses of radiotherapy and chemotherapy agents. Second, this study is a retrospective study, which might lead to a potential selection bias in the participants. Third, causes of death may be subject to misclassification ascertained from death certificates, and there was evidence indicating that causes on death certificates about CVM may be overestimated [49].

## 5. Conclusions

In summary, GEP-NEN patients were found to have a significantly increased risk of CVM in contrast to the general population. Patients, who were Black, non-Hispanic, male, unmarried; lived in the Midwest region; with an age at diagnosis ≥65, diagnosed between 2000 and 2004, Grade I/II, subtype of NET, localized SEER stage, primary site of small intestine, no surgery, no chemotherapy, no radiotherapy; lower educational level and higher household income, had a significantly higher CVM risk. Among the six causes of CVD, diseases of heart, hypertension without heart disease, cerebrovascular diseases, and other diseases of arteries, arterioles, and capillaries led to significantly elevated SMRs, and the CM rate of diseases of heart was the highest. Our findings suggested that after the diagnosis of GEP-NENs, patients should be screened for CVD in a timely manner and undergo more extensive control of modifiable risk factors for CVM. It also provided critical insights into how GEP-NEN patients should be followed up and counseled for relevant health risks. Additionally, further research is needed to understand the underlying mechanisms and to develop preventative and surveillance strategies for CVD in GEP-NEN patients.

## Figures and Tables

**Table 1:** Baseline features and standardized mortality ratios of cardiovascular mortality in patients with GEP-NENs.

		Observed deaths (%)	Expected deaths	SMR (95% CI)	Excess risk per 10,000	Persons (%)	Person years at risk
Total		1598 (100.0)	1335.17	1.20 (1.14-1.26)	12.63	42027 (100.0)	208158.81
Age at diagnosis	≤39	19 (1.2)	5.94	3.20 (1.93-4.99)	7.04	3438 (8.2)	18551.09
	40-44	22 (1.4)	11.93	1.84 (1.16-2.79)	7.20	2375 (5.7)	13975.98
	45-49	42 (2.6)	28.08	1.50 (1.08-2.02)	6.82	3610 (8.6)	20403.40
	50-54	85 (5.3)	80.47	1.06 (0.84-1.31)	1.15	7152 (17.0)	39442.59
	55-59	120 (7.5)	98.91	1.21 (1.01-1.45)	6.62	5925 (14.1)	31881.72
	60-64	148 (9.3)	125.91	1.18 (0.99-1.38)	8.20	5472 (13.0)	26923.19
	65-69	216 (13.5)	160.04	1.35 (1.18-1.54)	25.18	4859 (11.6)	22225.34
	70-74	248 (15.5)	196.42	1.26 (1.11-1.43)	32.69	3614 (8.6)	15780.00
	75-79	245 (15.3)	243.41	1.01 (0.88-1.14)	1.49	2736 (6.5)	10680.86
	80-84	249 (15.6)	239.02	1.04 (0.92-1.18)	17.09	1759 (4.2)	5837.49
	85+	204 (12.8)	145.04	1.41 (1.22-1.61)	239.97	1087 (2.6)	2457.14
Attained age	≤39	7 (0.4)	1.75	4.00 (1.61-8.24)	4.71	1964 (4.6)	11150.06
	40-44	15 (0.9)	4.18	3.59 (2.01-5.92)	11.66	1349 (3.2)	9279.41
	45-49	24 (1.5)	12.04	1.99 (1.28-2.97)	8.10	2279 (5.4)	14764.25
	50-54	54 (3.4)	36.68	1.47 (1.11-1.92)	6.44	4192 (10.0)	26890.15
	55-59	85 (5.3)	69.65	1.22 (0.97-1.51)	4.61	5628 (13.4)	33270.87
	60-64	117 (7.3)	100.68	1.16 (0.96-1.39)	5.13	6135 (14.6)	31835.12
	65-69	160 (10.0)	126.93	1.26 (1.07-1.47)	12.12	6208 (14.8)	27279.73
	70-74	219 (13.7)	154.03	1.42 (1.24-1.62)	30.87	4832 (11.5)	21043.16
	75-79	243 (15.2)	187.69	1.29 (1.14-1.47)	36.37	3927 (9.3)	15210.00
	80-84	245 (15.3)	223.75	1.09 (0.96-1.24)	21.06	2775 (6.6)	10086.80
	85+	429 (26.8)	417.80	1.03 (0.93-1.13)	15.24	2738 (6.5)	7349.26
Race	White	1163 (72.8)	1009.42	1.15 (1.09-1.22)	10.01	31176 (74.2)	153405.55
	Black	354 (22.2)	265.91	1.33 (1.20-1.48)	23.74	7411 (17.6)	37101.01
	Other	81 (5.1)	59.84	1.35 (1.07-1.68)	11.99	3440 (8.2)	17652.26
Hispanic origin	Non-Hispanic	1486 (93.0)	1228.03	1.21 (1.15-1.27)	13.93	37063 (88.2)	185214.91
	Hispanic	112 (7.0)	107.14	1.05 (0.86-1.26)	2.12	4964 (11.8)	22943.89
Gender	Male	807 (50.5)	705.59	1.14 (1.07-1.23)	10.00	20746 (49.4)	101446.47
	Female	791 (49.5)	629.58	1.26 (1.17-1.35)	15.13	21281 (50.6)	106712.34
Marital status	Married	720 (45.1)	721.21	1.00 (0.93-1.07)	-0.10	23712 (56.4)	123646.41
	Unmarried	734 (45.9)	487.62	1.51 (1.40-1.62)	39.29	14133 (33.6)	62709.18
	Unknown	144 (9.0)	126.33	1.14 (0.96-1.34)	8.10	4182 (10.0)	21803.21
Year of diagnosis	2000-2004	726 (45.4)	627.07	1.16 (1.08-1.25)	12.11	9143 (21.8)	81692.92
	2005-2009	567 (35.5)	468.04	1.21 (1.11-1.32)	12.66	12281 (29.2)	78179.06
	2010-2015	305 (19.1)	240.05	1.27 (1.13-1.42)	13.45	20603 (49.0)	48286.83
Latency (months)	0-1	137 (8.6)	37.67	3.64 (3.05-4.30)	147.80	2216 (5.3)	6720.98
	2-5	142 (8.9)	68.03	2.09 (1.76-2.46)	58.97	1762 (4.2)	12543.23
	6-11	125 (7.8)	94.40	1.32 (1.10-1.58)	17.73	1809 (4.3)	17263.08
	12-59	600 (37.5)	580.04	1.03 (0.95-1.12)	2.04	16636 (39.6)	97633.01
	60-119	434 (27.2)	410.15	1.06 (0.96-1.16)	4.16	11781 (28.0)	57388.46
	120+	160 (10.0)	144.88	1.10 (0.94-1.29)	9.10	7823 (18.6)	16610.05
Grade	I/II	362 (22.7)	329.90	1.10 (0.99-1.22)	5.51	16601 (39.5)	58249.56
	III/IV	71 (4.4)	53.70	1.32 (1.03-1.67)	29.16	3183 (7.6)	5932.23
	Unknown	1165 (72.9)	951.57	1.22 (1.15-1.30)	14.82	22243 (52.9)	143977.02
Education level	College level ≤25%	976 (61.1)	724.78	1.35 (1.26-1.43)	22.72	22671 (53.9)	110580.63
	College level >25%	622 (38.9)	609.86	1.02 (0.94-1.10)	1.25	19341 (46.0)	97508.26
Region	Midwest	203 (12.7)	168.23	1.21 (1.05-1.38)	16.08	4273 (10.2)	21621.17
	West	738 (46.2)	615.71	1.20 (1.11-1.29)	12.14	20510 (48.8)	100706.64
	South	433 (27.1)	319.32	1.36 (1.23-1.49)	22.40	10545 (25.1)	50748.01
	Northeast	224 (14.0)	231.91	0.97 (0.84-1.10)	-2.25	6699 (15.9)	35082.99
Mean household income	≤$50,000 USD	1129 (70.7)	898.10	1.26 (1.18-1.33)	17.09	27540 (65.5)	135144.75
	>$50,000 USD	469 (29.3)	436.54	1.07 (0.98-1.18)	4.45	14472 (34.4)	72944.13
Subtype	NEC	264 (16.5)	208.69	1.27 (1.12-1.43)	16.96	10558 (25.1)	32606.72
	NET	1334 (83.5)	1126.48	1.18 (1.12-1.25)	11.82	31469 (74.9)	175552.09
SEER stage	Localized	902 (56.4)	754.13	1.20 (1.12-1.28)	11.75	22388 (53.3)	125886.88
	Regional	316 (19.8)	292.87	1.08 (0.96-1.20)	5.93	7818 (18.6)	39005.99
	Distant	187 (11.7)	157.80	1.19 (1.02-1.37)	11.87	8546 (20.3)	24586.25
	Unstage	193 (12.1)	130.36	1.48 (1.28-1.70)	33.53	3275 (7.8)	18679.68
Primary site	Stomach	258 (16.1)	160.09	1.61 (1.42-1.82)	49.03	4287 (10.2)	19969.91
	Small intestine	683 (42.7)	529.00	1.29 (1.20-1.39)	25.57	11672 (27.8)	60214.91
	Appendix	58 (3.6)	46.12	1.26 (0.96-1.63)	8.78	3272 (7.8)	13535.66
	Colon	173 (10.8)	146.15	1.18 (1.01-1.37)	14.16	4256 (10.1)	18959.30
	Rectum	330 (20.7)	353.74	0.93 (0.83-1.04)	-3.11	12595 (30.0)	76355.76
	Pancreas	96 (6.0)	100.07	0.96 (0.78-1.17)	-2.13	5945 (14.1)	19123.27
Surgery	Yes	1145 (71.7)	1062.40	1.08 (1.02-1.14)	4.80	32265 (76.8)	172182.46
	No	438 (27.4)	261.23	1.68 (1.52-1.84)	52.03	9316 (22.2)	33977.78
	Unknown	15 (0.9)	11.54	1.30 (0.73-2.14)	17.31	446 (1.1)	1998.57
Chemotherapy	Yes	60 (3.8)	55.03	1.09 (0.83-1.40)	4.43	4337 (10.3)	11236.04
	No/unknown	1538 (96.2)	1280.14	1.20 (1.14-1.26)	13.09	37690 (89.7)	196922.77
Radiotherapy	Yes	13 (0.8)	12.69	1.02 (0.55-1.75)	1.22	985 (2.3)	2530.49
	No/unknown	1585 (99.2)	1322.48	1.20 (1.14-1.26)	12.77	41042 (97.7)	205628.32

I: Well differentiated, II: Moderately differentiated, III: Poorly differentiated, IV: Undifferentiated; Race: Other (American Indian & AK Native & Asian & Pacific Islander); Marital status: Unmarried (Single & Separated & Divorced & Widowed & Unmarried or Domestic Partner); Attained age was defined as the age of the patient at the time of death or end of follow-up. Abbreviation: SMR: standardized mortality ratio; CI: confidence interval; AER: absolute excess risk; NET: neuroendocrine tumor; NEC: neuroendocrine carcinoma.

**Table 2:** The standardized mortality ratios of all causes of cardiovascular mortality in patients with GEP-NENs.

CVD	Observed deaths (%)	Expected deaths	SMR (95% CI)	AER per 10,000
Total	1598 (100)	1335.17	1.20 (1.14-1.26)	12.63
Diseases of heart	1204 (75.3)	1018.14	1.18 (1.12-1.25)	8.93
Hypertension without heart disease	67 (4.2)	47.14	1.42 (1.10-1.81)	0.95
Cerebrovascular diseases	271 (16.9)	223.69	1.21 (1.07-1.36)	2.27
Atherosclerosis	9 (0.6)	12.13	0.74 (0.34-1.41)	-0.15
Aortic aneurysm and dissection	20 (1.2)	18.59	1.08 (0.66-1.66)	0.07
Other diseases of arteries, arterioles, capillaries	27 (1.7)	15.49	1.74 (1.15-2.54)	0.55

Abbreviation: CVD: cardiovascular disease; SMR: standardized mortality ratio; CI: confidence interval; AER: absolute excess risk.

**Table 3:** Cumulative mortality stratified by age at diagnosis and primary site at 200 months follow-up.

Characteristics		Cumulative morality of all causes of death			
		Primary cancer	Cardiovascular disease	Other cancer	Other noncancer diseases
Age at diagnosis (years)	<50	9.58	3.10	10.84	6.94
	50-64	10.63	5.47	15.13	10.12
	65-79	14.90	16.32	23.44	21.71
	≥80	21.94	25.89	22.36	26.78
Primary site	Stomach	11.41	12.56	15.49	20.75
	Small intestine	4.74	13.26	25.12	18.42
	Appendix	15.79	4.84	10.99	10.52
	Colon	25.37	9.09	18.96	10.76
	Rectum	2.92	7.65	10.35	10.07
	Pancreas	41.81	4.12	17.20	11.47

**Table 4:** Multivariate competing risk analysis for predictors of cardiovascular mortality in patients with GEP-NENs.

Characteristics		Adjusted HR	95% CI	*P*
Age at diagnosis (years)	<65	Ref		
	≥65	4.799	4.313-5.341	<0.001
Race	White	Ref		
	Black	1.307	1.160-1.472	<0.001
	Other	0.784	0.626-0.982	0.034
Hispanic origin	Hispanic	Ref		
	Non-Hispanic	1.370	1.137-1.651	<0.001
Gender	Male	Ref		
	Female	0.790	0.717-0.869	<0.001
Marital status	Married	Ref		
	Unmarried	1.562	1.410-1.173	<0.001
	Unknown	1.171	0.984-1.394	0.076
Year of diagnosis	2000-2004	Ref		
	2005-2009	0.798	0.717-0.888	<0.0001
	2010-2015	0.575	0.502-0.659	<0.0001
Grade	I/II	Ref		
	III/IV	0.701	0.533-0.923	0.011
	Unknown	1.116	0.985-1.265	0.085
Education level	College level ≤25%	Ref		
	College level >25%	0.798	0.706-0.902	<0.001
Mean household income	≤$50,000 USD	Ref		
	>$50,000 USD	1.024	0.895-1.171	0.73
Region	West	Ref		
	Midwest	1.011	0.870-1.176	0.88
	South	0.943	0.834-1.065	0.34
	Northeast	0.813	0.699-0.945	<0.01
Subtype	NET	Ref		
	NEC	0.984	0.842-1.150	0.84
SEER stage	Localized	Ref		
	Regional	0.815	0.714-0.931	<0.01
	Distant	0.456	0.382-0.544	<0.001
	Unstage	0.990	0.840-1.167	0.9
Primary site	Stomach	Ref		
	Small intestine	1.055	0.911-1.222	0.48
	Appendix	0.698	0.531-0.918	0.01
	Colon	0.844	0.698-1.020	0.079
	Rectum	0.550	0.468-0.646	<0.001
	Pancreas	0.506	0.401-0.638	<0.001
Surgery	Yes	Ref		
	No/unknown	1.346	1.188-1.519	<0.001
Chemotherapy	Yes	Ref		
	No/unknown	1.610	1.220-2.125	<0.001
Radiotherapy	Yes	Ref		
	No/unknown	1.514	0.881-2.602	0.13

I: Well differentiated; II: Moderately differentiated; III: Poorly differentiated; IV: Undifferentiated; Race: Other (American Indian & AK Native & Asian & Pacific Islander); Marital status: Unmarried (Single & Separated & Divorced & Widowed & Unmarried or Domestic Partner). Abbreviation: HR: hazard ratio; CI: confidence interval; NET: neuroendocrine tumor; NEC: neuroendocrine carcinoma.

## Data Availability

The datasets analyzed in this study are available in the SEER repository and can be obtained from: https://seer.cancer.gov/data/.
